# Current state of knowledge of basic life support in health professionals of the largest city in Pakistan: a cross-sectional study

**DOI:** 10.1186/s12913-019-4676-y

**Published:** 2019-11-21

**Authors:** Babar Irfan, Ibrahim Zahid, Muhammad Sharjeel Khan, Omar Abdul Aziz Khan, Shayan Zaidi, Safia Awan, Sobia Bilal, Omar Irfan

**Affiliations:** 1 Jinnah Postgraduate Medical Center, Karachi, Pakistan; 2 Dow University of Health Sciences, Karachi, Pakistan; 3 Altamash Institute of Dental Medicine, Karachi, Pakistan; 4 Jinnah Sindh Medical University, Karachi, Pakistan; 5 Department of Medicine, Aga Khan University Hospital, Karachi, Pakistan; 6 Faculty School of Dentistry, Lead IMU-Quit Smoking Service, International Medical University, Kuala Lumpur, Malaysia; 7 Peter Gilgan Center of Research and Learning, Hospital for Sick Children, Toronto, Canada

**Keywords:** BLS knowledge, ACLS, Cardiopulmonary resuscitation, Doctors, Dentists, Nurses, Education, Training

## Abstract

**Background:**

Basic Life Support (BLS) is the recognition of sudden cardiac arrest and activation of the emergency response system, followed by resuscitation, and rapid defibrillation. According to WHO, Pakistan has one of the highest mortality rates from accidental deaths therefore assessment and comparison of BLS knowledge in health professionals is crucial. We thereby aim to assess and compare the knowledge of BLS in doctors, dentists and nurses.

**Methods:**

A multi-centric cross-sectional survey was conducted in Karachi at different institutions belonging to the private as well as government sector from January to March 2018. We used a structured questionnaire which was adapted from pretested questionnaires that have been used previously in similar studies. Descriptive statistics were analyzed using SPSS v22.0, where adequate knowledge was taken as a score of at least 50%. *P* < 0.05 was considered as significant. Logistic regression was used to identify the factors affecting the knowledge regarding BLS in health care professionals.

**Results:**

The responders consisted of 140 doctors, nurses and dentists each. Only one individual (dentist) received a full score of 100%. In total, 58.3% of the population had inadequate knowledge. Average scores of doctors, dentists and nurses were 53.5, 43.3 and 38.4% respectively. Doctors, participants with prior training in BLS and those with 6 to 10 years after graduation were found to be a significant predictor of adequate knowledge, on multivariate analysis.

**Conclusion:**

Even though knowledge of BLS in doctors is better than that of dentists and nurses, overall knowledge of health care professionals is extremely poor. Present study highlights the need for a structured training of BLS for health care workers.

## Background

Basic Life Support is the recognition of sudden cardiac arrest (SCA) followed by activation of emergency response system, early cardiopulmonary resuscitation (CPR), and rapid defibrillation with an automated external defibrillator (AED) [1]. For decreasing the mortality rate and increasing survival ratio it is crucial for a health care provider to have a firm grip on basic cardiac life support knowledge and practices [2]. Globally, about 92% out-of-hospital cardiac arrest subjects lose their lives due to limited availability of CPR facilities. One of the leading causes of death and disability worldwide is out-of-hospital cardiac arrest (OHCA) and it contributes to as high as 10% of total mortality in developing countries [3]. According to the World Health Organization (WHO), Pakistan has some of the highest mortality rates from injuries such as road traffic accidents and accidental deaths with a recent review reported 146,000 deaths and 2.8 million injuries from road traffic accidents alone [4].

Health care professionals encounter such emergency situations very often so they should have sufficient knowledge of BLS [5]. Apart from doctors and nurses, dental practitioners as a part of health care professionals also encounter life-threatening medical emergencies. A study [6] found that during the 12-month study period about two-thirds of dentists faced at least one emergency. Moreover, there are some reports showing that during dental treatment patients died due to cardiopulmonary arrest [7].

A study in India found the knowledge of BLS to be extremely poor in their study on medical, dental and nursing students, doctors and nurses [8]. In another study from South Africa, poor knowledge and skills of medical practitioners in basic resuscitation were reported [9]. In 2009 medical students from Karachi, Pakistan were evaluated and more than half of them were found to have no knowledge of BLS; it was concluded that prior training in BLS would improve the knowledge and its application [10]. On the other hand, a study on junior doctors from UK found them to be not capable enough to perform effective resuscitation even when life support training was being provided [11]. All these examples from different regions indicate a poor state of knowledge of BLS in health professionals and undergraduate students of related fields. Yet, no study has been conducted to compare the knowledge of BLS in 3 vital categories of health care that is, doctors, dentist and nurses, in Pakistan, and evaluate the factors affecting their awareness.

Therefore, the aim of the study was to assess the knowledge of BLS in doctors, dentists and nurses and identify the factors affecting the knowledge regarding BLS in health care professionals. This would highlight the deficits in the current curriculum in these fields and help in guiding future planning of BLS programs in Pakistan. After this study, we hope that all aspects of BLS training for health personnel be improved, standardized and made more accessible.

## Methods

A multi-center cross-sectional study was conducted in five institutions of Karachi from January to March 2018. The institutions included National Institute of Child Health, Altamash Institute of Dental Medicine, Dow University Hospital, Dr. Ruth K.M Pfau Civil Hospital Karachi, School of Nursing and Out-patient department of Jinnah Hospital, Karachi, ensuring that health care professionals are covered from a greater part of the city to minimize selection bias. The questionnaire was administered to the concerned staff on duty in different departments of the hospital based on non-probability convenience sampling. Based on the assumption that 15.2% of the study sample had adequate knowledge about BLS in a developing country [12], and taking a 5% margin of error and 90% confidence level, the calculated sample turned out to be 140. Therefore, 140 dentists, doctors and nurses each were included in our study which was designed for a similar setting, taking the total sample size to 420. The incomplete response forms were excluded from the final count of sample and data collection was conducted until 140 questionnaires were collected for each of the categories.

Prior to data collection, written informed consent was sought and participation in the study was voluntary. The survey was conducted after approval from Ethical Review Committee of the concerned institutions. The survey was divided into two sections; demographics and knowledge of BLS. Data were collected through a questionnaire (see Additional file 1) which is according to current AHA/ERC guidelines of 2015 [13]. We used a structured questionnaire which was adapted from pretested questionnaires that have been used previously in similar studies in India [8, 14, 15] and in Saudi Arabia [16]. The questionnaire was then assessed by carrying out a pilot study among the experienced medical fraternity (*n* = 50), and the necessary corrections were made accordingly after consultation with an emergency care specialist. Each question in the survey had the same weightage.

Data were entered and analyzed through IBM-Statistical Package for the Social Sciences (SPSS) version 22. A descriptive analysis is presented as number and percentages. Total score was categorized into two; less than 50% and more than or equal to 50%. Association between knowledge score were assessed by using the Chi-square test or Fisher exact test where appropriate. For post hoc tests following a Chi-Square, we use Bonferroni Adjustment; this adjustment is used to counteract the problem of Type I Error that occurs when multiple comparisons are made. Odds Ratios (OR) and their 95% Confidence Intervals (CI) were estimated using Logistic Regression, with knowledge score as an outcome. All *p*-values were two sided and considered as statistically significant if *P* < 0.05.

## Results

Demographic data of the study is as shown in Table 1. Most of the participants were female (63.6%), in the 21–30 age group (72.1%), graduated less than 5 years ago (71.9%), with no prior formal training in BLS (52.9%). Out of the 420 participants, 212 (50.5%) were Intern or resident, and 50 (11.9%) were clinical faculty.

**Table 1:** Demographics of 420 participants

Characteristic	Frequency	Percentage (%)
Gender		
Male	153	36.4
Female	267	63.6
Age		
21–30	303	72.1
31–40	86	20.5
41–50	27	6.4
51–60	04	1.0
Education		
Doctors	140	33.3
Dentists	140	33.3
Nurses	140	33.3
Designation		
Intern	97	23.1
Resident	115	27.4
Faculty	50	11.9
Others	158	37.6
Years since graduation		
0	01	0.2
0–5	302	71.9
6–10	82	19.5
11–15	30	7.1
16–20	05	1.2
Prior training in BLS		
Yes	198	47.1
No	222	52.9

Table 2 displays the responses of the 20 questions with the total correct and incorrect answers along with percentages of the 3 categories individually. The responses were statistically significant in all questions except questions pertaining to location for chest compression in infants, rescue breathing in infants, depth of compression in adults and neonates during chest compressions, chest compression and ventilation ratio in newborn, and first response for symptoms of choking (*p* < 0.05). Most notably, majority failed to correctly answer the depth of compressions in adults, the method of giving rescue breaths in infants, chest compression and ventilation ratio in newborn and first response following choking (correct answers< 20%).

**Table 2:** Number of correct and incorrect responses

	Doctors	Dentists	Nurses	Total	*P*-Value
1. What is the abbreviation of “BLS”?					
Correct	136(97.1%)	135(96.4%)	122(87.1%)*	393(93.6%)	0.001
Incorrect	4(2.9%)	5(3.6%)	18(12.9%)	27(6.4%)	
2. When you find someone unresponsive in the middle of the road, what will be your first response? (Note: You are alone there)					
Correct	94(67.1%)	83(59.3%)	71(50.7%)	248(59.0%)	0.02
Incorrect	46(32.9%)	57(40.7%)	69(49.3%)	172(41.0%)	
3. If you confirm somebody is not responding to you even after shaking and shouting at him, what will be your immediate action?					
Correct	65(46.4%)*	36(25.7%)	35(25.0%)*	136(32.4%)	< 0.001
Incorrect	75(53.6%)	104(74.3%)	105(75.0%)	284(67.6%)	
4. What is the location for chest compression?					
Correct	102(72.9%*	70(50.0%)	63(45.0%)	235(56.0%)	< 0.001
Incorrect	38(27.1%)	70(50.0%)	77(55.0%)	185(44.0%)	
5. What is the location for chest compression in infants?					
Correct	55(39.3%)	68(48.6%)	52(37.1%)	175(41.7%)	0.11
Incorrect	85(60.7%)	72(51.4%)	88(62.9%)	245(58.3%)	
6. If you do not want to give mouth-to-mouth CPR, the following can be done EXCEPT					
Correct	90(64.3%)	76(54.3%)	60(42.9%)*	226(53.8%)	0.002
Incorrect	50(35.7%)	64(45.7%)	80(57.1%)	194(46.2%)	
7. How do you give rescue breathing in infants?					
Correct	21(15.0%)	31(22.1%)	19(13.6%)	71(16.9%)	0.12
Incorrect	119(85.0%)	109(77.9%)	121(86.4%)	349(83.1%)	
8. Depth of compression in adults during CPR					
Correct	18(12.9%)	20(14.3%)	11(7.9%)	49(11.7%)	0.21
Incorrect	122(87.1%)	120(85.7%)	129(92.1%)	371(88.3%)	
9. Depth of compression in Children during CPR					
Correct	54(38.6%)	35(25.0%)	49(35.0%)	138(32.9%)	0.04
Incorrect	86(61.4%)	105(75.0%)	91(65.0%)	282(67.1%)	
10. Depth of compression in neonates during CPR					
Correct	53(37.9%)	49(35.0%)	54(38.6%)	156(37.1%)	0.80
Incorrect	87(62.1%)	91(65.0%)	86(61.4%)	264(62.9%)	
11. Rate of chest compression in adult and Children during CPR					
Correct	81(57.9%)	64(45.7%)	61(43.6%)	206(49.0%)	0.03
Incorrect	59(42.1%)	76(54.3%)	79(56.4%)	214(51.0%)	
12. Ratio of CPR, single rescuer in adult is					
Correct	97(69.3%)*	69(49.3%)	57(40.7%)*	223(53.1%)	< 0.001
Incorrect	43(30.7%)	71(50.7%)	83(59.3%)	197(46.9%)	
13. In a new born the chest compression and ventilation ratio is					
Correct	20(14.3%)	18(12.9%)	28(20.0%)	66(15.7%)	0.22
Incorrect	120(85.7%)	122(87.1%)	112(80.0%)	354(84.3%)	
14. What does abbreviation AED stand for?					
Correct	87(62.1%)*	69(49.3%)	52(37.1%)*	208(49.5%)	< 0.001
Incorrect	53(37.9%)	71(50.7%)	88(62.9%)	212(50.5%)	
15. What does abbreviation EMS stand for?					
Correct	111(79.3%*	91(65.0%)	62(44.3%)*	264(62.9%)	< 0.001
Incorrect	29(20.7%)	49(35.0%)	78(55.7%)	156(37.1%)	
16. If you and your friend are having food in a canteen and suddenly your friend starts expressing symptoms of choking, what will be your first response?					
Correct	40(28.6%)	31(22.1%)	32(22.9%)	103(24.5%)	0.39
Incorrect	100(71.4%)	109(77.9%)	108(77.1%)	317(75.5%)	
17. You are witnessing an infant who suddenly started choking while he was playing with the toy, you have confirmed that he is unable to cry (or) cough, what will be your first response?					
Correct	104(74.3%*	83(59.3%)	74(52.9%)	261(62.1%)	0.001
Incorrect	36(25.7%)	57(40.7%)	66(47.1%)	159(37.9%)	
18. You are witnessing an adult unresponsive victim who has been submerged in fresh water and just removed from it. He has spontaneous breathing, but he is unresponsive. What is the first step?					
Correct	40(28.6%)*	11(7.9%)	26(18.6%)	77(18.3%)	< 0.001
Incorrect	100(71.4%)	129(92.1%)	114(81.4%)	343(81.7%)	
19. You noticed that your colleague has suddenly developed slurring of speech and weakness of right upper limb. Which one of the following can be done?					
Correct	109(77.9%*	67(47.9%)	61(43.6%)*	237(56.4%)*	< 0.001
Incorrect	31(22.1%)	73(52.1%)	79(56.4%)	183(43.6%)	
20. A 50-year-old gentleman with retrosternal chest discomfort, profuse sweating and vomiting. What is next?					
Correct	122(87.1%*	107(76.4%)	85(60.7%)*	314(74.8%)	< 0.001
Incorrect	18(12.9%)	33(23.6%)	55(39.3%)	106(25.2%)	

*p-value for post hoc chi-square test: 0.05/6 = 0.0083

In total, 58.3% of the individuals who answered the questionnaire received less than 50% marks, corresponding to inadequate knowledge. Only one individual –a dentistry resident- was able to achieve a full score of 100%. Interns had a mean score of 42.8 ± 14.2 whilst their academic seniors, the residents had an almost similar mean score of 43.1 ± 16.4. Faculty scored slightly higher with an average score of 49.4 ± 13.4. Students and medical and house officers who were all grouped in one category had an average score of 46.6 ± 16.0, (*p* value = 0.02). On average, the scores of doctors, dentists and nurses were 53.5 ± 14.2, 43.3 ± 13.4 and 38.4 ± 15.0 respectively, (*p* value< 0.001).

Univariate and multivariate analyses of the data are shown in Table 3. Collectively, being a doctor (odds ratio (OR), 5.89, 95% CI: (2.91 to 11.89), a faculty member (odds ratio (OR), 3.49, 95% CI: (1.51 to 8.05), before and having attended a BLS course (odds ratio (OR), 2.19, 95% CI: (1.34 to 3.56), was significantly associated with greater knowledge. While years of education graduated 6–10 years (odds ratio (OR), 0.46, 95% CI: (0.24 to 0.88) have a protective effect on knowledge score.

**Table 3:** Univariate and multivariate logistic regression

Characteristic	Odds Ratio[95% CI]	*P*-value
Univariate analysis		
Age		
21–30	Reference	
31–40	0.88[0.54–1.43]	0.61
41–50	0.79[0.35–1.79]	0.57
51–60	1.34[0.18–9.70]	0.76
Gender		
Female	Reference	
Male	0.75[0.49–1.12]	0.16
Designation		
Intern	Reference	
Resident	1.02[0.58–1.80]	0.92
Faculty	2.0[1.0–4.01]	0.04*
Others	1.63[0.96–2.74]	0.06
BLS course		
No	Reference	
Yes	3.43[2.28–5.15]	< 0.001*
Education		
Nurses	Reference	
Doctors	6.89[4.06–11.70]	< 0.001*
Dentists	1.81[1.07–3.07]	0.02*
Years since graduation		
0–5	Reference	
6–10	0.36[0.20–0.63]	< 0.001*
> 10	1.26[0.62–2.55]	0.50
Multivariate analysis		
Education		
Nurses	Reference	
Doctors	5.89[2.91–11.89]	< 0.001*
Dentists	1.30[0.69–2.45]	0.41
Years		
0–5	Reference	
6–10	0.46[0.24–0.88]	0.02*
> 10	1.80[0.79–4.10]	0.16
BLS course		
No	Reference	
Yes	2.19[1.34–3.56]	0.002*
Designation		
Intern	Reference	
Resident	2.41[1.19–4.90]	0.01*
Faculty	3.49[1.51–8.05]	0.003*
Others	1.85[1.00–3.41]	0.04*

Hosmer-Lemeshow goodness-of-fit; 0.47; χ2 = 6.59

*CI* Confidence interval; *P<0.05

## Discussion

The level of awareness regarding BLS among doctors, dentists and nurses in a tertiary care setting, in general, was found to be inadequate. This is more alarming as members of the health care community are expected to have knowledge and the skills to be able to perform CPR [17], and they are looked upon as the leaders when a needful situation arises [18].

Unfortunately, the doctors, nurses and dentists did not perform well when their knowledge regarding BLS was tested in our study, with only 67.1% of the doctors, 35% dentists and 22.9% of the nurses having an adequate amount of knowledge regarding BLS (score ≥ 50%) with their average scores being along the same pattern, as mentioned previously. While a similar study from South India [8] observed similar orderliness when adjusted according to scores, another study from Nepal [2] found the mean score of nurses to be greater than that of the dentists. The former study had only 15.2% of the participants scoring greater than 50% [8], however, in our study much greater proportion (41.7%) was able to achieve the same. Our findings are in line with several studies which observed poor knowledge among medical students [19] and health care professionals while simultaneously proving that medical interns had greater knowledge regarding BLS as compared to dental interns [20]. A study conducted in North Kerala, which is said to have close to 100% literacy rate with its health care sector reaching international standards, with a study sample which has the mean score to be 44.5% [21] which corresponds to the overall mean score of knowledge of our study (45.1%). This was further corroborated by other studies in India where the mean score was found to be 41.6% [3], 36.05% [22] and Nepal where it was 44% [2]. It is noteworthy that only less than 2% of our participants were able to exceed the 80% mark set by AHA, analogous to the 4.3% quoted in Kerala [21].

About half of the individuals were able to correctly identify the location (56.0%) and number of chest compressions (49.0%) required in an adult per minute and their ratio to breaths (53.1%). This was corroborated by the findings of a study conducted in South India [3] and Karantaka [23]. There are studies who found a negative relationship of physician’s experience with their knowledge of BLS, like the one by Zamir et al. [24] and Nambiar et al. [21]. Our study concluded that a time period of 6 to 10 years since graduation and being a faculty member was significantly associated with adequate knowledge regarding BLS (Table 3), which is possible due to higher number of training courses and the chances of increased amount of ‘hands on’ real life experiences with greater years of practice. On multivariate analysis, after adjusting for other demographic factors, a prior course in BLS was significantly associated with increased amount of knowledge, which corresponds to the findings of previous literature [12, 25, 26]. While the number and duration since the last course taken was not assessed in our study, it has been found in previous studies that retraining enhances a person’s knowledge and skills related to BLS [27, 28]. Cooper et al. [5] further proved that provision of a short Immediate Life Support (ILS) course following 6 months after a BLS course significantly improves the knowledge and skills of people.

There can be several causes behind the disappointing figures from our survey. Nonetheless, factors which restrict the health care professionals from receiving regular BLS training are compelling. Hectic residency schedules and scarcity of resources act as a major barrier. Lack of passion to learn resuscitation during internship and residency training, limited number of BLS programs being offered, lack of assessment tools following a training course, decreased encounter with relevant patients, difficulty in retaining the knowledge and increasing number of professionals as compared to the inadequate slots in training programs are only a few limitations to adequate knowledge of BLS. A study demonstrate that cardiopulmonary arrest can occur in some of emergency cases in dental practice if they are not managed properly [29]. Starc et al. demonstrated how first year medical students had satisfactory BLS skills when assessed shortly after a course, thereby allowing the deduction that the more recent the training the greater the skillfulness [30]. This calls for refresher training courses in addition to the mandatory BLS course taken once around graduation, to allow for persistent expertise in the skill. This is the case in our Country where post-graduation career lacks any of such comprehensive courses which can refresh BLS skills. Therefore, knowledge of BLS in these health professionals is important.

BLS training should be made compulsory and must be included in the curriculum of all undergraduate and post graduate training schools [31], as appropriate training of BLS improves survival rates and following resuscitation of cardiac arrest patients [32] and such courses with hands on practice are essential for the betterment of CPR outcomes. It has been shown in many studies that knowledge and skills of CPR performance decrease following 6 months after training [33, 34] however the performance ameliorates when nurses are retrained in the same [35]. While adequate knowledge of CPR has been correlated with better performance, some studies have found that correct execution may be more dependent on regular practice through motor skills than on retention of guidelines [36–38].

We suggest, based on prior literature, BLS courses should not just be made mandatory for all health care professionals before entering their program but also, regular workshops should be arranged to reinforce the training throughout their career with recommendations to attend a course at least once in 2 years. BLS certified organizations should be established to cater to the increasing demand of health care professionals being produced in the country to arrange innovative hands-on workshops for the candidates with real life scenarios, and already established workshops should aim to target the same audience following 6 months after a course to plan a refresher course.

One major strength of this study is that we compared the 3 categories of health professionals from five institutes of Karachi, Pakistan. However, the study has some limitations as well; it was a questionnaire based study and the skill set and improvising capabilities of the individuals added to their intellect could not be measured. Hence, it is recommended to conduct further studies on a larger scale from various cities of Pakistan to address the nation as a whole. Moreover, the sample size used is not large enough as compared to previous similar studies. The study included only the participants who completed the whole questionnaire in the final analysis. Any incomplete forms were discarded. This technique likely prevented us to gauge the response rate and the non-reporting bias assessment. 

## Conclusions

In conclusion, this study indicates that knowledge of BLS among health professionals from medicine, nursing and dentistry in Karachi is poor and needs to be improved. The deficits in the knowledge of BLS in health care professionals of the largest and most populous city of Pakistan is a huge concern. We suggest a strict accreditation program to the undergraduate curriculum, along with regular reassessments along the career of the health professionals.

## Supplementary information

**Additional file 1.** Questionnaire.

## Data Availability

The datasets used and/or analyzed during the current study are available from the corresponding author on reasonable request.

## Abbreviations

AED: Automated external defibrillator
BLS: Basic Life Support
CI: Confidence Interval
CPR: Cardiopulmonary resuscitation
ILS: Immediate Life Support
OHCA: Out-of-hospital cardiac arrest
OR: Odds ratio
SCA: Sudden cardiac arrest
SPSS: Statistical Package for the Social Sciences
WHO: World Health Organization
