# Supplementary material for: Current state of knowledge of basic life support in health professionals of the largest city in Pakistan: a cross-sectional study
Source: BMC Health Serv Res. 2019 Nov 21;19:865. doi: 10.1186/s12913-019-4676-y (PMC6868838; doi:10.1186/s12913-019-4676-y)
Supplement: Supplementary file 1 — Additional file 1. Questionnaire. [file 12913_2019_4676_MOESM1_ESM.docx]

**Questionnaire**

| 1. What is the abbreviation of “BLS”?    1. Best Life Support    2. Basic Life Support    3. Basic Lung Support    4. Basic Life Services 2. When you find someone unresponsive in the middle of the road, what will be your first response? (Note: You are alone there)    1. Open airway    2. Start chest compression    3. Look for safety    4. Give two breathings 3. If you confirm somebody is not responding to you even after shaking and shouting at him, what will be your immediate action?    1. Start CPR    2. Activate EMS    3. Put him in recovery position    4. Observe 4. What is the location for chest compression?    1. Left side of the chest    2. Right side of the chest    3. Mid chest    4. Xiphisternum 5. What is the location for chest compression in infants?    1. One finger breadth below the nipple line    2. One finger breadth above the nipple line    3. At the intermammary line    4. At Xiphisternum 6. If you do not want to give mouth-to-mouth CPR, the following can be done *EXCEPT*    1. Mouth-mask ventilation and chest compression    2. Chest compression only    3. Bag mask ventilation with chest compression    4. No CPR 7. How do you give rescue breathing in infants?    1. Mouth-to-mouth with nose pinched    2. Mouth-to-mouth and nose    3. Mouth-to-nose only    4. Mouth-to-mouth without nose pinched 8. Depth of compression in adults during CPR    1. 1½ – 2 inches    2. 2½ – 3 inches    3. 1 – 1½ inches    4. ½ – 1 inch 9. Depth of compression in Children during CPR    1. 1½ – 2 inches    2. 2½ – 3 inches    3. One-half to one-third depth of chest    4. ½ – 1 CM 10. Depth of compression in neonates during CPR     1. 1½ – 2 inches     2. 2½ – 3 inches     3. ½ – 1 CM     4. One-half to one-third depth of chest 11. Rate of chest compression in adult and Children during CPR     1. 100 / min     2. 120 / min     3. 80 / min     4. 70 / min 12. Ratio of CPR, single rescuer in adult is     1. 15:2     2. 5:1     3. 30:2     4. 15:1 13. In a new born the chest compression and ventilation ratio is     1. 15:2     2. 5:1     3. 30:2     4. 3:1 14. What does abbreviation AED stand for?     1. Automated External Defibrillator     2. Automated Electrical Defibrillator     3. Advanced Electrical Defibrillator     4. Advanced External Defibrillator 15. What does abbreviation EMS stand for?     1. Effective Medical Services     2. Emergency Management Services     3. Emergency Medical Services     4. External Medical Support 16. If you and your friend are having food in a canteen and suddenly your friend starts expressing symptoms of choking, what will be your first response?     1. Give abdominal thrusts     2. Give chest compression     3. Confirm foreign body aspiration by talking to him     4. Give back blows 17. You are witnessing an infant who suddenly started choking while he was playing with the toy, you have confirmed that he is unable to cry (or) cough, what will be your first response?     1. Start CPR immediately     2. Try to remove the suspected foreign body by blind finger sweeping technique     3. Back blows and chest compression of five cycles each then open the mouth and remove foreign body only when it is seen     4. Give water to the infant 18. You are witnessing an adult unresponsive victim who has been submerged in fresh water and just removed from it. He has spontaneous breathing, but he is unresponsive. What is the first step?     1. CPR for two minutes and inform EMS     2. CPR for one minute and inform EMS     3. Compress the abdomen to remove the water     4. Keep him in recovery position 19. You noticed that your colleague has suddenly developed slurring of speech and weakness of right upper limb. Which one of the following can be done?     1. Offer him some drinks, probably hypoglycemia     2. Possibly stroke, get him to the nearest clinic     3. Possibly stroke, he may require thrombolysis and hence activate emergency medical services     4. May be due to sleep deprivation, make him sleep. 20. A 50-year-old gentleman with retrosternal chest discomfort, profuse sweating and vomiting. What is next?     1. Probably myocardial infarction, hence activates EMS, give an aspirin tablet and allow him to rest     2. Probably acid peptic disease, give antacid and Ranitidine     3. Probably indigestion, hence give soda     4. Take him by walk to the nearest clinic. |
| --- |

**Answer key:**

1 ( B ) 5 ( A ) 9 ( C ) 13 ( D ) 17 ( C )

2 ( C ) 6 ( D ) 10( D ) 14 ( A ) 18 ( D )

3 ( B ) 7 ( B ) 11( A ) 15 ( C ) 19 ( C )

4 ( C ) 8 ( A ) 12( C ) 16 ( C ) 20 ( A )
